# Vital signs in pediatric oncology patients assessed by continuous recording with a wearable device, NCT04134429

**DOI:** 10.1038/s41597-022-01182-z

**Published:** 2022-03-17

**Authors:** Marion Haemmerli, Roland A. Ammann, Jochen Roessler, Christa Koenig, Eva Brack

**Affiliations:** 1 Division of Pediatric Hematology/Oncology, Department of Pediatrics, Inselspital, Bern University Hospital, University of Bern, Bern, Switzerland; 2 Kinderaerzte KurWerk, Burgdorf, Switzerland

**Keywords:** Paediatric research, Fever

## Abstract

Pediatric patients with cancer are at high risk for severe infections. Changes in vital signs, triggered by infections, may be detected earlier by continuous recording with a wearable device than with discrete measurements. This prospective, observational single-center feasibility study consecutively recruited pediatric patients undergoing chemotherapy for cancer. The WD Everion® was used for 14 days in each of the 20 patients on study to continuously record vital signs. Nine different vital signs and health indicators derived from them, plus six quality scores. This resulted in 274 study days (6576 hours) with 85’854 measuring points, which are a total of 772’686 measurements of vital signs and health indicators, plus 515’124 quality scores. Additionally, non-WD data like side effects, acceptability of the WD and effort for investigators were collected. In this manuscript, we present the methods of acquisition and explanations to the complete data set, which have been made publically available on open access and which can be used to study feasibility of continuous multi-parameter recording of vital signs by a WD.

## Abstract (summary)

**Table:** 

Measurement(s)	Vital signs continiously
Technology Type(s)	Wearable Device
Factor Type(s)	Core temperature â€¢ Galvanic skin response â€¢ Health score â€¢ Heart rate â€¢ Heart rate variability â€¢ Oxygen saturation â€¢ Skin temperature â€¢ Skin blood perfusion
Sample Characteristic - Organism	Homo sapiens

## Background & Summary

In pediatric patients with cancer, neutropenia is a common side effect of chemotherapy. During chemotherapy-induced neutropenia, fever is often the only clinical detectable sign of a potentially life-threatening infection. Therefore, fever in neutropenia is treated as an emergency, leading to hospitalization and start of intravenous empirical broad-spectrum antibiotics^1^. Delay of diagnosis may result in more intensive treatment, more adverse events and increased mortality^2–4^. With the exception of fever, published clinical decision rules helping to distinguish between severe and less severe infections^5–7^ do not rely on vital signs.

In the adult medical setting, noninvasive wearable devices (WDs) have become widely available and allow continuous measurement of patients’ vital signs^8–10^ (https://biofourmis.com), including core temperature^11,12^ nearly under any condition. Further, during the Covid-19 pandemic, WDs have extensively been used to monitor affected patients^13–15^.

However, in children continuous monitoring is more difficult as most of the devices are either not approved in children, or not appropriate to wear due to their size, weight or mode of body attachment. Further patient acceptance might be more difficult in children compared to adults. Despite these difficulties, few pediatric studies have been published for example in healthy children with wrist-worn WDs to monitor heart rate^16^ and physical activity^17,18^. Further, several studies have assessed physical activity in pediatric patients undergoing chemotherapy with WDs^19–22^

So far, not many pediatric studies have reported on patters in vital signs preceding infections, for example heart-rate variability^23,24^. Using a WD therefore opens the possibility to identify online, i.e., without relevant delay from detection to processing, vital sign patterns that might be used as an additional diagnostic tool or a tool predicting imminent fever or infection in the near future^25^. In addition, it may help to distinguish patients at lower versus higher risk for severe infections by providing more detailed information, and a more exact representation of the patient’s health status than discrete measurements.

The data described here^25^ has been collected during a prospective, single-center observational study (November 30, 2019 to January 13, 2020) in pediatric patients between ≥1 month to <18 years, undergoing chemotherapy for cancer.

The aim of this study was to assess the feasibility of continuous multi-parameter recording of vital signs with the WD Everion® in pediatric patients undergoing chemotherapy for cancer and to compare these recorded vital signs with discrete measurements.

At administration, patients’ basic characteristics, like the skin type according to the Fitzpatrick Scale^26^, were assessed. During the study period of 14 days, patients were asked to wear the WD as often as possible and to fill out a daily case report form (CRF) on time of charging, connection with the application, physical activities, potential WD side effects, hospitalization, contacts with the investigators and results of optional discrete temperature measurements at home. Information on discrete measurements taken for clinical reasons during hospitalization or outpatient visit, was retrieved from patient charts^27^. A follow up interview on acceptability and usability of the WD further took place at the end of the study period.

In total, 20 patients were included in the study. As one patient withdrew informed consent (IC) after eight study days, vital signs and health indicators, plus data quality were collected at a total of 274 days (6576 hours). This resulted in 85’854 measuring points, which are a total of 772’686 measurements of vital signs and health indicators, plus 515’124 quality scores. Quality of recorded heart rate was good during 3992 of 6576 (61%) hours, poor during 300 (5%) hours and no data was recorded during 2284 (35%) hours. During this time, 46 episodes of physical activity, three episodes of fever (one in neutropenia) and one local skin infection were reported.

All analytical results on the recorded vital signs, their quality, the discrete measurements and feasibility-related outcomes have been published elsewhere^27^.

These data can be used to study feasibility of continuous multi-parameter recording of vital signs by a WD. They provide information about compliance, acceptability and usability of a WD like Everion®, as well as information about the data quality. If feasibility is given, WDs can help to detect infections earlier than discrete measurements and therefore result in less intensive treatment, less adverse events and decreased mortality, as well as helping to distinguish between severe and less severe infections.

The data can further be used to study different vital signs and signals in pediatric patients undergoing chemotherapy for any malignancy. They may as well be used for comparison with vital signs from healthy children without chemotherapy or for pattern search. Moreover, they can be used for comparison studies in children with other diseases unrelated to cancer. In addition we believe they can be used for comparison with vital signs from adults with or without chemotherapy for cancer.

We assume merging these data with other data sets is feasible.

## Methods

These methods are expanded versions of descriptions in our corresponding analytical work^27^.

### Study design

The data described here^25^ had been collected during the study “Continuous recording of vital signs with a wearable device in pediatric patients undergoing chemotherapy for cancer – an operational feasibility study”^27^. This prospective, single-center observational study was performed at the Division of Pediatric Hematology/Oncology, Department of Pediatrics, Inselspital, Bern University Hospital, University of Bern, Switzerland. The Division of Pediatric Hematology/Oncology in Bern gives tertiary care for a population of approximately one million inhabitants. The department is divided into an inpatient unit with 8 beds, as well as a large outpatient unit. This enables this division on the one hand to treat about 40 newly diagnosed pediatric patients with all types of malignancies per year, and on the other hand to perform myeloablative chemotherapy followed by autologous stem cell transplantation for most of Switzerland, which has a population of about 8.5 million inhabitants.

The study was conducted in accordance with the Declaration of Helsinki and the principles of Good Clinical Practice. Approval was granted by the local Ethics Committees (Ethikkommission der Universitätskinderkliniken Bern) prior to patient recruitment and the trial has been registered at www.clinicaltrials.com (NCT04134429). Written IC was obtained from the patients, if able to judge, and from their legal guardians prior to study entry. All additionally gave written consent that the coded study data will be published. Participation in this observational study did not influence any diagnostic nor therapeutic decisions^27^.

### Patients

To enter the study, the patients had to fulfill all inclusion criteria: age between 1 month and 17.99 years at time of recruitment, myelosuppressive chemotherapy treatment for any malignancy, expected to last ≥1 month at time of recruitment; or at least one cycle of myeloablative therapy before autologous hematopoietic stem cell transplantation, and written IC from patients and/or their parents. Patients with local skin disease prohibiting wearing the WD and patients without written IC were excluded. Of 40 Patients potentially eligible, 20 (with a median age of 6 years, range 2 to 16; 9 patients <6 years) participated in the study. One patient withdrew IC after eight study days. One patient had remaining time of chemotherapy below 1 month but covered the entire study duration of 14 days. So, this patient remained on study despite the formal violation of an inclusion criterion^27^. Patients on study, were mainly outpatients, however some patients required hospitalization during their study period and so were also treated as inpatients.

Patients were screened for eligibility and consecutively recruited by a study investigator or treating physician. No specific screening procedures except verifying inclusion and exclusion criteria were needed. Patients were assessed during a routine outpatient visit or during hospitalization. A description of the patient characteristics, clinical data as well as the reported outcomes can be found in Table 1.

**Table 1:** Patient characteristics and outcome.

Description	Amount
**Age** years median (range)	6 (2–16)
<6 years	9
**Sex**	
Female	9
Male	11
**Type of malignancy**	
ALL	12
Other hematologic malignancies	2
CNS	3
Solid tumor	3
**Location of patient during study**	
Inpatient (days)	33
Outpatient (days)	13
At home (days)	228
**Amount of days on which device was worn**	274
**Amount of measurements of vital signs (n)**	772686
**Amount of quality scores (n)**	515124

### Wearable device on study & data management

The WD used in this study was the Everion® VSM-1 device by Biovotion (now Biofourmis), Zurich, Switzerland (firmware version, 3.15.0) (Biofourmis. (https://support.biofourmis.com/hc/en-us/categories/201377109-Everion-Device, 2020)), which has not yet been studied or validated in usage by children with chemotherapy for cancer.

There are nine signals recorded by the WD in this study (Table 2). The WD assigns a quality score (range 0–100%) to six of the vital signs: heart rate, heart rate variability, oxygen saturation, respiration rate, core temperature and skin blood perfusion. For heart rate and skin blood perfusion the quality score is the same. According to the manufacturer a quality score of ≥50 indicates good data quality meeting the accuracy criteria.

**Table 2:** Data recorded by the WD Everion^®^.

Recorded data	Unit	Range	Quality score	Description
Core temperature	°C	21–46	Core temperature quality	Temperature of the body
Galvanic skin response	kOhm	0–5	—	A measurement of electrodermal activity as an index of sympathetic nervous system activity – sensitive measure for emotional arousal (Biofourmis. (https://support.biofourmis.com/hc/en-us/articles/213838709-Electrodermal-activity-galvanic-skin-response, 2020))
Health score	—	0–100%	—	Shows moment-to-moment balance between bodily stress and recovery. Stress during the day = body resources deplete, leisure time (mostly during night sleep) = resources replenish.
				In perfect balance, one would arrive at the same health level after one full wake-sleep cycle (Biofourmis. (https://support.biofourmis.com/hc/en-us/articles/360011425079-Behavior-Metrics, 2020))
Heart rate	bpm	30–240	Heart rate/skin blood perfusion quality	How many heartbeats a person has per minute
Heart rate variability	ms	0–255	Heart rate variability quality	Interval variation between two heart beats
Oxygen saturation	%	60–100	Oxygen saturation quality	Oxygen saturation in the arterial blood
Respiration rate	bps	2–60	Respiration rate quality	How many breaths a person takes per minute
Skin temperature	°C	0–60	—	Temperature of the skin
Skin blood perfusion	—	0–5	Heart rate/skin blood perfusion quality	Blood flow to the capillary bed

Everion® generates signals as vital signs or scores at a frequency of one per second. For this study, only aggregates, calculated every 3 min from the primary measurements, were collected. For the six vital signs with a quality score assigned, the aggregate measurement over the three past minutes is a quality-weighted average of the corresponding 180 primary per-second measurements. All primary measurements with a quality score >50 are used and then weighted by subtracting 30 of the assigned quality score. E.g., a measurement with a quality score of 40 is not used for calculating the aggregate, a measurement with a quality score of 50 is used with a weight of 20, and a measurement with a quality score of 90 is used with a weight of 60. For the three vital signs without quality score assignment, the calculation is different: For skin temperature and galvanic skin response, the aggregate is the median of all primary measurements within the three past minutes. For health score, the last measurement is used as aggregate. Aggregate quality assignments are the average of all quality assignments >50, if more than 10% of quality assignments are >50. Otherwise, it is the average of all quality assignments <50. (Data Glossary by Biovotion, revision 1, generation date: 28-Mar-2019, unpublished).

Calculated aggregates were transferred via Bluetooth® to an AndroidTM mobile phone application (AugmentStudy App, version 1.3.5), which displayed the connection status with the Internet and the WD and the WD’s battery status. The application automatically uploaded coded data to a cloud-based password-protected dashboard (Everion® Dashboard, version 6.0), to which only the study team had access. The entire data processing (data in transport and data stored) was encrypted by Biovotion. Data was retrieved by the researchers from the dashboard as device-specific json files using json scripts and then imported into R^28^ via Microsoft® Excel. After completion of analysis and storage of WD data in a local research database, data were irreversible deleted from the cloud-based dashboard. WD data was then edited in Microsoft® Excel and finally shared on Figshare 10.6084/m9.figshare.13471338.v3^25^.

### Study procedures & non-WD data

Patients’ basic characteristics were collected at administration. All requirements for conducting the study were made by one of the investigators: distributing the WD, a suitable mobile phone if needed and a set of paper CRFs. Participants were instructed to fill out daily CRFs, where they reported about time of charging, connection with the mobile phone application, physical activities, potential WD side effects, if the WD was judged as wearable this day, reasons not to wear the WD, and, if applicable, results of discrete temperature measurements performed for clinical reasons at home. Daily data checks were performed, and participants were called in case of missing or implausible WD data. If applicable, possible reasons for missing data were discussed and instructions given. Calls were replaced by personal visits in hospitalized patients or during outpatient visits. Information about the type of contacts and actions taken were documented by the investigator on a daily report forms, as well as information on localization of the patient (hospitalization, visit on outpatient ward, at home) and on diagnosis of fever or infection. Within 3 days after the study period, a follow-up interview was done to assess acceptability and usability of the WD. All non-WD data, be it noted by the investigators or by the participants, was collected on paper CRFs or report forms and stored using Redcap® electronic data capture tools^29^. From there the data was transferred into Microsoft® Excel, where it was processed and from which the figshare document was created.

### Data coverage of this study

This study collected data on vital signs, continuously recorded by the WD Everion® and non-WD data as patients’ basic characteristics, side effects, usability and acceptability from each patient, collected during administration, on daily CRFs and during the follow-up interview.

## Data Records

A single data record resulted from this study, containing a total of 23 single data files (Table 3): One file contains all the non-WD data of all 20 patients on the administration, follow-up and outcomes of the daily CRF, which were noted by the investigators or reported by the participants (File Non_WD_data_all_patients). Detailed information on variable specification is included in a corresponding readme file (File Non_WD_data_all_patients_Readme).

**Table 3:** Overview.

Observations	Time covered	Center	Data source	Data
Patients	11.2019–01.2020	Bern	Administration, daily CRF, follow-up interview, patients’ charts	Non_WD_data_all_patients
Patients	11.2019–01.2020	Bern	WD Everion®	WD_data.patient_xx

The remaining 21 files (File WD_data.patient_xx), one per patient, where xx refers to a patient-specific random number, contain the data of the vital signs recorded by the WD, plus another readme file, explaining all the variables used in there (File WD_data.patient_xx_Readme).

All the obtained data are published on Figshare: 10.6084/m9.figshare.13471338.v3^25^.

## Technical Validation

### Reduction of recruitment bias

In order to reduce recruitment bias, all eligible patients from the cancer center were consecutively invited for study participation, regardless their diagnosis, age and gender. Distribution of age, gender and type of malignancy of patients screened, refusing IC, not asked for IC, and those included in the study were comparable^27^. Furthermore, the participating study patients correspond to a representative group of pediatric cancer patients regarding distribution of gender, age and diagnosis.

### Data collection and increasing data reliability

Within the setting of a prospective clinical study, the data presented here was extracted by an experienced pediatric oncology fellow from the administration, the daily CRFs and the follow-up interview questionnaires. Further, results from discrete measurements taken for clinical reasons during outpatient visits or hospitalization were extracted from the patients’ charts. All the information was entered into a Redcap® database^29^ and checked for plausibility.

Raw data on vital signs was retrieved from the Everion® dashboard as device-specific json files and processed in R into patient-specific restricted data files before analysis, using the following steps: After sorting the information according to timestamps, double lines of information with identical timestamps were deleted (randomly distributed lines in all device files, 5 to 75 lines per device, corresponding to 0.1 to 1.2% of data). Then, to match the CET (Central European Time) used for the study, there was 1 hour added to the GMT (Greenwich Mean Time) based WD stamps and the absolute dates of timestamps were replaced by study day (1 to 14). The device-specific data files where then transferred into patient-specific data files, where applicable. And finally, information outside the study period and about variables not studied have been deleted^27^.

### Anonymization procedure

Before publication, data were anonymized in order to comply with Swiss research legislation. The date of study start for each patient, considered to be a potential identifier, was replaced by day 1. Further days were correspondingly calculated from day 1 on. The real time of the day was expressed as a fraction of the day. Data on diagnosis, gender and age stayed in the dataset, considering that this is a prospective study and that all patients and their legal guardians gave IC to the publication of these data.

## Data Availability

Microsoft® Excel was used to enter, store and quality check the collected data. Json file was used only to download WD data from the dashboard and was provided by Everion®. This file is not needed to access, use or process the here provided data sets. The R script used for quality checks of the provided data is available on Figshare^25^.
